# Turtle Carapace Anomalies: The Roles of Genetic Diversity and Environment

**DOI:** 10.1371/journal.pone.0018714

**Published:** 2011-04-12

**Authors:** Guillermo Velo-Antón, C. Guilherme Becker, Adolfo Cordero-Rivera

**Affiliations:** 1 Department of Ecology and Evolutionary Biology, Cornell University, Ithaca, New York, United States of America; 2 Grupo de Ecoloxía Evolutiva e da Conservación, Departamento de Ecoloxía e Bioloxía Animal, Universidade de Vigo, E.U.E. Forestal, Campus Universitario, Pontevedra, Spain; University of Sao Paulo, Brazil

## Abstract

**Background:**

Phenotypic anomalies are common in wild populations and multiple genetic, biotic and abiotic factors might contribute to their formation. Turtles are excellent models for the study of developmental instability because anomalies are easily detected in the form of malformations, additions, or reductions in the number of scutes or scales.

**Methodology/Principal Findings:**

In this study, we integrated field observations, manipulative experiments, and climatic and genetic approaches to investigate the origin of carapace scute anomalies across Iberian populations of the European pond turtle, *Emys orbicularis*. The proportion of anomalous individuals varied from 3% to 69% in local populations, with increasing frequency of anomalies in northern regions. We found no significant effect of climatic and soil moisture, or climatic temperature on the occurrence of anomalies. However, lower genetic diversity and inbreeding were good predictors of the prevalence of scute anomalies among populations. Both decreasing genetic diversity and increasing proportion of anomalous individuals in northern parts of the Iberian distribution may be linked to recolonization events from the Southern Pleistocene refugium.

**Conclusions/Significance:**

Overall, our results suggest that developmental instability in turtle carapace formation might be caused, at least in part, by genetic factors, although the influence of environmental factors affecting the developmental stability of turtle carapace cannot be ruled out. Further studies of the effects of environmental factors, pollutants and heritability of anomalies would be useful to better understand the complex origin of anomalies in natural populations.

## Introduction

The occurrence of anomalies, malformations, or asymmetries in wild animals may serve as an indicator of developmental instability, a variable negatively correlated with fitness [1], [2]. Developmental anomalies can result from several factors which have the potential to decrease individual fitness, such as genetic disorders, or biotic and abiotic factors (e.g. parasites, chemicals or other environmental conditions) that affect developing embryos. Thus, individuals from stressed populations usually display higher levels of anomalies than those from non-stressed populations [3].

**Figure 1:**
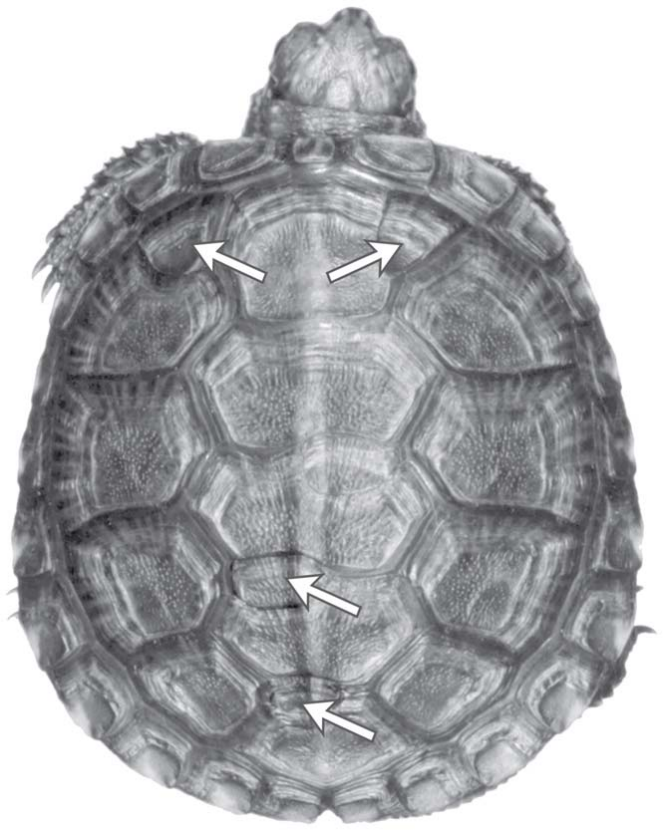
Example of common carapace anomalies in a hatchling obtained from our experimental incubation survey. The individual bellow has four extra-scutes: between first vertebral and both first costal scutes; and between third-fourth and fourth-fifth vertebral scutes (the standard shell pattern contains five vertebral scutes and four costal scutes on both sides).

Most reptiles, and particularly turtles, are excellent models for the study of developmental instability because anomalies are easily detected in the form of malformations, additions, or reductions in the number of scutes or scales (Figure 1). Three non-exclusive sources have been proposed as the main causes of scute or scale anomalies in reptiles: i) temperature and moisture constraints during incubation [4], [5], ii) damaging effects of pollution [6], [7], and iii) loss of genetic diversity in bottlenecked or inbred populations [8], [9], [10].

Most studies reporting the occurrence of carapace anomalies have focused on incubation conditions (temperature and moisture). High number of scute anomalies has been detected in previous experimental studies when eggs were exposed to partial drying during critical developmental stages (e.g., at the middle stage of developmental in *Chrysemys picta* and *Chelydra serpentina*, [5]), or under extreme conditions of temperature [11]; although other studies excluded moisture as a significant factor on scute anomaly formation [12], [13]. In addition, some pollutants (e.g., polychlorinated aromatic hydrocarbons and organochlorines such as DDT) have been identified as teratogenic agents in vertebrates [14], [15], [16]. For the common snapping turtle *Chelydra serpentina* chemicals including organochlorine pesticides, polychlorinated biphenyls and mercury were identified as main causes of developmental abnormalities in both embryos and hatchlings [7], [17].

In addition to these environmental factors, developmental stability is also influenced by intrinsic genetic characteristics: the loss of genetic diversity has a significant effect upon the occurrence of scale anomalies in the dice snake, *Natrix tessellata* [10], and high homozygosity has been linked to developmental anomalies [8]. A disproportionate occurrence of malformations has been attributed to a phylogenetic predisposition to anomalous development in certain species. For instance, a comprehensive study of 118 turtle species from seven families documented anomalies variation among species, and higher levels of carapace anomalies in aquatic species than in semi-aquatic and terrestrial species [18].

Our understanding of the complexity of causes of anomalies in nature would benefit from studies that integrate multiple potential causative factors. However, measuring the influence of several potential extrinsic and intrinsic causes is a complex task. Not surprisingly, integrative research on the relative roles of genetic diversity and environmental factors (moisture/temperature constraints and pollution) in determining turtle shell anomalies is largely unexplored.

The European pond turtle (*Emys orbicularis*, Linnaeus 1758) is a freshwater species from the family Emydidae. It has a widespread distribution ranging from East and Central Europe, to the Mediterranean countries and the North of Africa [19]. Previous genetic surveys of Iberian populations identified three subspecies, one with widespread distribution (*E. o. fritzjuergenobsti*; [20]), and two others that colonized northeastern Iberia after the last glacial period [21]. Extant Iberian populations are small, particularly in northern Iberia, and face many threats such as habitat fragmentation, pollution, and illegal collecting for the pet trade [22]. The prevalence of scute anomalies in the carapace of endangered northwest Iberian populations is high, likely due to those multiple stressors [23]. We surveyed the presence of scute anomalies for each captured individual throughout Iberian populations [24] and confirmed an increasing trend of anomalies from southern to northern populations. However, the causes of these anomalies and potential sources for this development instability are still unknown.

In this study we investigated the relative roles of environmental stressors and genetic diversity in shaping this geographic trend of carapace scute anomalies in Iberian populations of *E. o. fritzjuergenobsti*. We focused on the effects of (i) water potential during egg incubation period; (ii) latitude and climatic variables (temperature and precipitation); and (iii) genetic diversity, inbreeding and bottleneck events. By integrating experimental, climatic, and genetic data across Iberian populations, we infer the likely causes of shell anomaly proportions among populations and discuss other potential factors that may affect the developmental stability of turtle carapace.

## Materials and Methods

### Sampling design and study populations

**Figure 2:**
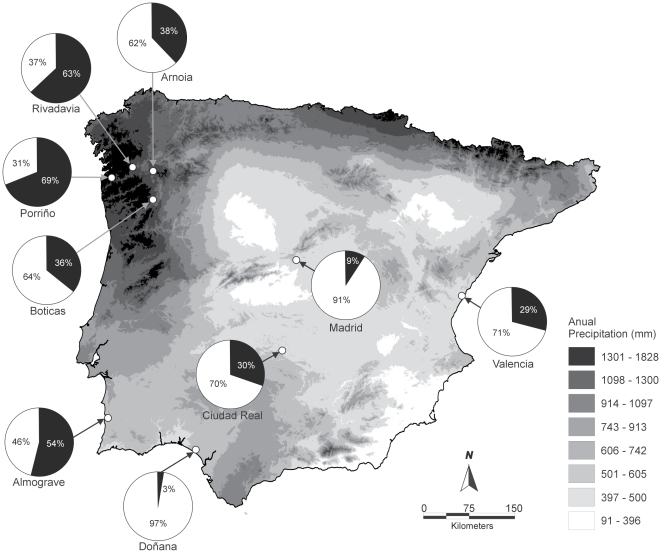
Annual precipitation for the Iberian peninsula and percentage of anomalies (black) found for the nine Iberian populations of *E. orbicularis*.

We first considered a wide geographic scale to investigate potential genetic and environmental factors contributing to anomalies using microsatellite and climatic datasets across nine Iberian populations (Figure 2). We obtained environmental and phenotype data (Table 1) for nine Iberian populations surveyed in a previous study [24]. For the genetic approach we used a dataset analyzed in a previous study on the genetic structure of Iberian populations of *E. orbicularis* [20]. We excluded four out of 13 populations surveyed in previous studies based on small sample size (Castroverde, N = 11; Salamanca N = 7, and Zamora N = 18), and lack of genetic data (Castroverde and Salamanca). The population of Menorca was discarded because it belongs to a different subspecies introduced to the Balearic islands. Moreover, samples from Zamora belonged to several relatively isolated streams, and thus, they might not constitute one population. We added Ribadavia population to the original genetic dataset in this study by genotyping 35 individuals. Approximately 100 *µl* of blood was obtained from the occipital venous sinus of each animal. Ribadavia is a key population to understand the causes of the origin of anomalies because it is the northernmost sampled Iberian population and the second highest anomalous population (Fig. 2).

**Table 1:** Populations, localities and datasets used in this study: anomalies within populations (*N*, sample size; number of anomalies for each individual; mean number of anomalies, *Mean*; and percentage of anomalies), genetic diversity values (*N*, sample size; observed heterozygosity, *H_O_*; allelic richness, *A-R*; and relatedness, *r*), and environmental data (minimum temperature, *Tmin* (°C); maximum temperature, *Tmax* (°C); and precipitation, *Prec* (mm), from April to October on fifty years time period 1950–2000).

			Anomalies									Genetic diversity				Environmental data		
Population	Locality	Latitude	N	0	1	2	3	4	5	Mean	%	N	H_O_	A-R	r	Tmin	Tmax	Prec
Porriño	Gandaras de Budiño (NW)	42.17	166	52	45	38	25	3	3	1.34	69	30	0.65	3.34	0.30	13.8	21.7	62.6
Ribadavia	Ribadavia (NW)	42.28	43	16	12	7	5	3	0	1.23	63	35	0.64	2.58	0.52	13.2	22.8	54.2
Arnoia	Baños de Molgas (NW)	42.18	74	46	16	10	1	1	0	0.58	38	32	0.66	3.63	0.17	11.0	22.2	52.5
Boticas	Vila Real (NW)	41.68	42	27	5	8	1	0	1	0.69	36	28	0.69	3.73	0.19	11.0	22.8	51.2
Madrid	El Escorial (C)	40.40	32	29	2	1	0	0	0	0.13	9	31	0.76	4.11	0.16	11.3	23.5	32.2
Ciudad Real	Abenójar (C)	38.98	23	16	5	2	0	0	0	0.39	30	23	0.74	4.03	0.19	13.2	28.1	27.4
Valencia	Sagunto, Burriana & Moro (CE)	39.47	55	39	11	5	0	0	0	0.38	29	25	0.73	3.87	0.25	17.0	25.3	31.7
Almograve	Alentejo(SW)	37.65	26	12	6	4	4	0	0	1.00	54	27	0.75	3.99	0.23	14.6	25.1	17.8
Doñana	Doñana National Park (S)	37.26	34	33	1	0	0	0	0	0.03	3	36	0.82	4.29	0.11	17.1	26.4	17.7

Populations are located in Northwestern (NW), central (C), central-Eastern (CE), Southwestern (SW) and Southern (S) regions of Iberia.

### Genetic diversity and past demographic analyses

Genomic DNA extraction, PCR conditions and genotyping followed protocols in [20]. Given the low evolutionary rate of the mitochondrial genome of turtles [25] and the lack of haplotypic diversity for D-loop, Cytb and ND4 mtDNA regions within Iberian *Emys* populations [20], [21], genetic diversity and demographic aspects were investigated using microsatellite data. Our final dataset contained 267 individuals genotyped at seven microsatellite markers across nine populations. On average 30 individuals were included per population (Table 1).

We assessed Hardy-Weinberg equilibrium (HWE) by applying a Markov chain exact test [26] for each locus in each population. Linkage disequilibrium for each pair of loci in each population was performed using GENEPOP on the web (http://www.wbiomed.curtin.edu.au/genepop/). For both tests, significance was assessed after sequential Bonferroni correction for multiple tests [27]. Genetic diversity of *E. o. fritzjuergenobsti* was assessed by estimating observed heterozygosity (H_O_) over all loci using the software GENETIX [28] and allelic richness (AR) [29]. The program hp-rare [29], which uses a rarefaction method for estimating how many alleles are expected in a sample of specified size, was used to compute estimates of unbiased allelic richness. We also estimated genetic relatedness (*r*) within-populations, [30] with the software GENALEX v.6 [31]. We estimated mean pairwise within populations relatedness and compared those values to confidence intervals obtained by bootstrapping over genotypes within populations (1000 replicates). We used the coefficient of relatedness as a measure of inbreeding in each population (r = 0 among unrelated individuals; r = 0.5 among full sibs; r = 1 among identical twins).

To test for genetic evidence of recent population bottlenecks, we used three different microsatellite-based approaches. First, we used BOTTLENECK [32] implemented by Cornuet and Luikart [33]. This method tests for recent reductions of effective population size by considering that alleles are generally lost faster than heterozygosity and assuming population at mutation-drift equilibrium [34]. Thus, recently bottlenecked populations will display an excess of heterozygosity based on the observed number of alleles. Following the recommendations of the program for limited sample size, we used the Wilcoxon sign-rank test under two mutation models: the stepwise mutation model (SMM) and the two-phased model (TPM) with 70% stepwise mutation model, and 1 million replicates. Second, we used a qualitative descriptor of the allele frequency distribution (mode-shift indicator); recently bottlenecked populations should depart from a standard L-shape because alleles with low frequency have disappeared as a consequence of the loss of genetic diversity [35]. Third, we used the M-ratio test implemented by Garza and Williamson [36], which uses the statistic *M* (the ratio of the number of alleles to range in allele size) to detect reductions in effective population size for a sample of microsatellite loci. This method considers that quantitative diversity (frequency of alleles and total number of alleles, *k*) is reduced more quickly than spatial diversity (distance between alleles and overall range in allele size, *r*) in bottlenecked populations. Three parameters are needed for the M-ratio test: effective population size (θ), percentage of mutations greater than one step (Δg), and average size of a non one-step mutation (r). Given the absence of reliable estimates of these parameters, we performed a sensitivity analysis of θ and Δg using a range of more and less conservative values found in the literature (Table 2).

**Table 2:** Bottleneck results for each population based on heterozygosity excess (*He*), allele frequency analyses (Mode shift) and M-ratio tests.

Population	He (p-values)	Mode-shift	M-ratio
Porriño	0.4063	L-shaped	0.80
Ribadavia	0.5313	L-shaped	***0.76*** 1 ^,^ 2
Arnoia	0.1484	L-shaped	***0.90*** 1
Boticas	***0.0039***	L-shaped	***0.80*** 1
Madrid	***0.0039***	L-shaped	***0.78*** 1 ^,^ 2
Valencia	0.4063	L-shaped	0.80
Ciudad Real	0.3438	L-shaped	0.89
Almograve	0.4063	L-shaped	***0.80*** 1
Doñana	***0.0078***	L-shaped	0.90

Three parameter sets used for M-ratios were:

1 p<0.05 (θ = 0.5 Δg = 2.8 r = 0.1),

2 p<0.05 (θ = 1 Δg = 2.8 r = 0.1), and

3 p<0.05 (θ = 5 Δg = 3.5 r = 0.1). Significant values are expressed in bold.

To further investigate the heritability of anomalies we split the dataset into two subsets (anomalous and non-anomalous) and estimated, for each subset and population, mean pairwise relatedness, *r*. Estimates of 95% confidence intervals around mean relatedness estimates were generated using 1000 bootstrap replicates. We used the northernmost Iberian populations (Porriño, Ourense, Ribadavia and Boticas), which belong to the same genetic deme [20] and differ from other genetic groups occurring in the Iberian Peninsula, ensuring a better estimate of relatedness among individuals from these populations.

To survey for a possible genetic basis for anomaly occurrence, we tested for statistical associations between microsatellite allele frequencies and anomalous phenotypic individuals (defined as anomalous or not anomalous) using the program STRAT v.1.0 [37]. Microsatellite loci can be linked to functionally-important genomic regions [38] and thus one or more of the seven microsatellites genotyped in this study might be linked to the genes or gene complexes controlling the expression of scute anomalies on the turtle carapace. We excluded from this analysis three populations without individual phenotypic information (Almograve, Ciudad Real and Valencia).

### Environmental analyses

The individuals surveyed in this study were from juveniles up to old adults (from one up to 40–60 years probably) and thus we used available temperature and moisture information for the last 50 years in all studied populations. Current information on soil moisture and temperature would not reflect the environmental conditions experienced during embryos' development in the individuals surveyed. On the contrary, and based on current knowledge of potential environmental factors influencing the occurrence of scute anomalies in reptiles, we used climatic layers for maximum temperature, minimum temperature, and precipitation from June to August (when embryonic development occurs in Iberian populations) [39], [40] to estimate the influence of those three climatic factors on anomaly patterns found across Iberian populations of *E. orbicularis*. We obtained these data as environmental layers at 30 s (∼1 km) resolution from WorldClim 1.4 [41] (dataset based on fifty years time period; 1950–2000). We made a circular landscape of 3 km diameter centred on each sampling site and extracted all climatic variables based on ten equidistant measurements within each locality. Climatic analyses were done in ArcView 9.3 [42].

Previous structure analyses indicated that each population in our dataset represents an independent genetic deme [20]. Even so, we tested for non-random spatial distribution of mean anomalies across Iberian populations using spatial autocorrelation analyses [43] to reassure independency among sampled populations. After assuring random spatial distribution of anomalies [mean anomalies (Moran's I index of spatial autocorrelation = 0.18, Z = 1.85 SD, P = 0.10) and proportion of anomalies (I = 0.17, Z = 1.77 SD, P = 0.10)] we performed simple linear regressions to evaluate the effect of genetic diversity indices and environmental variables (latitude, minimum temperature, maximum temperature, and precipitation) on the mean number of anomalies in sampled populations [44].

We ran Generalized Linear Models - GLMs (normal distribution and identity link) including one genetic index in turn and the four environmental variables to estimate the relative strength of each factor on mean anomalies. Subsequently, we avoided multicollinearity among cross-correlated environmental variables by running analyses of Principal Components (PC) including correlated environmental variables. When two or more variables in GLMs are highly correlated, they both convey essentially the same information. All environmental variables in our study are cross-correlated (e.g., latitude and precipitation are highly correlated: *r* = −0.986, *P*<0.0001). When it happens, the variables are collinear and cannot be considered independent. Principal Component Analysis is a widely used method for reducing multicollinearity when running GLMs. In this method, the original variables are transformed into a new set of orthogonal or uncorrelated variables called PCs. This transformation ranks the new orthogonal variables in order of their importance and then involves eliminating PCs with low cumulative explanatory power to effect a reduction in variance. After eliminating the least important PCs, we performed GLMs of the response variable against the first and most important PC. In this study, the PC1 of the four mentioned environmental factors had a cumulative percentage of 77.98%.

We ran GLMs to estimate the relative importance of genetic indices and environmental factors explaining mean anomalies in *E. orbicularis* (Table S1). In each of the three models, we included one genetic index (Ho, A-R, or *r*) and the PC1 of environmental factors as explanatory variables, allowing the interaction between them. We used sample size as weight estimation in all models to correct for slightly different sampling effort among populations. Analyses were run using JMP 8.0 (SAS 2009).

### Experimental incubation

We incubated eggs from 30 females of *E. orbicularis* from four northwestern Iberian populations (Porriño, Arnoia, Ribadavia and Boticas) during 2006–2007 (Table 1; Fig. 2). These northern populations are isolated and relatively small (with the exception of Boticas) and their sex-ratio is male-biased (Cordero Rivera et al. unpublished data), which make difficult capturing egg-bearing females. We did not include clutches from further populations due to the limitations of transporting individuals of threatened species throughout long distances. All females were captured using baited traps and individually marked by filing notches in their marginal scutes to allow individual recognition at consecutive samplings during the same season and successive years. We maintained females in independent terraria filled with soil until oviposition. Individuals were released at their respective collection localities. The normal number of carapace scutes in turtles is five vertebrals, four pairs of costals, and 12 pairs of marginals. Any deviation of vertebral or costal scute numbers or their pattern was quantified as an anomaly. Many studies on developmental stability have concentrated on measuring fluctuating asymmetry (FA), because deviations from perfect bilateral symmetry reflect environmental factors, given that the genetic factors are the same in both sides of the body [44]. Our study does not address FA but concentrates on the environmental or genetic effects leading to the formation of scute anomalies. In fact, anomalous individuals can be symmetric when they have lack or extra vertebral scutes, and when they show the same extra coastal scutes in both sides.

We incubated eggs in moistened vermiculite at constant temperature (28°C, the pivotal temperature for sex determination in *E. orbicularis*; [45]). To assess the influence of moisture on anomalies during the embryonic development, every clutch was split into two boxes with different substrate water potentials: −150 kPa (wet) and −600 kPa (dry) [46], [47]. To control for moisture during the incubation period we weighed each box without the lid, adding water drops to restore the initial weight if needed. To avoid gradient temperature effects we randomly placed boxes in the incubator and switched them inside the incubation chamber every week.

Once hatched, we calculated the proportion of successfully hatched eggs for every clutch (number of hatched eggs/number eggs of the clutch), and every treatment (number of hatched eggs/number of eggs in the treatment). We checked each hatchling for the presence of carapace anomalies, including a few embryos that died but whose carapace scutes were already formed. To test the effect of *treatment*, *year*, *population* and *maternal symmetry* on the proportion of anomalous hatchlings, and the proportion of eggs that hatched, we used a Generalized Linear Model (GLM) with binomial distribution and logit link. The effect of *treatment* on incubation duration and hatchling weight was further tested with an ANOVA, including *year* as a random factor and *population* as a fixed factor. Statistical analyses were performed with Genstat 12.

## Results

### Genetic diversity and estimates of population bottlenecks

One out of 63 χ-square tests showed significant deviation from Hardy-Weinberg equilibrium at the 95% confidence interval after sequential Bonferroni correction (D16 in Ribadavia population). As in the previous genetic study of Iberian populations, we found linkage disequilibrium (after sequential Bonferroni tests; p<0.05) between loci D88 and D114 (Porriño, Boticas and Ribadavia) and loci D88 and D51 (Doñana and Valencia). We found a latitudinal genetic trend across Iberian populations, with genetic diversity (measured as observed heterozygosity and allelic richness; Table 1) decreasing from southern (Doñana and Almograve) to northern populations (Porriño, Arnoia and Ribadavia), and relatedness values (*r*) increasing from Doñana (0.11) to Ribadavia (0.52). The Ribadavia population has the lowest genetic diversity and highest relatedness, with individuals in this population showing relatedness equivalent to that of full sibs.

The Wilcoxon signed-rank test in the BOTTLENECK analyses detected significant evidence of a recent bottleneck across the Iberian *Emys* distribution: northern (Boticas), central (Madrid), and southern population (Doñana), (p<0.01; see Table 2). However, the qualitative descriptor (L-shift indicator, implemented also in BOTTLENECK) failed to find evidence of recent bottlenecked populations. The analyses for bottleneck detection performed with M-ratio test found evidence of bottlenecks in five populations, when less restrictive parameters were used (θ = 0.5, Δg = 2.8); only two when the population size parameter was increased to 1, and none with conservative parameters (θ = 1, Δg = 2.8; see Table 2). Surprisingly, for the southernmost population, which has the highest genetic diversity (Table 1), clear evidence of a bottleneck was detected based on excess heterozygosity, but not with the M-ratio test.

**Figure 3:**
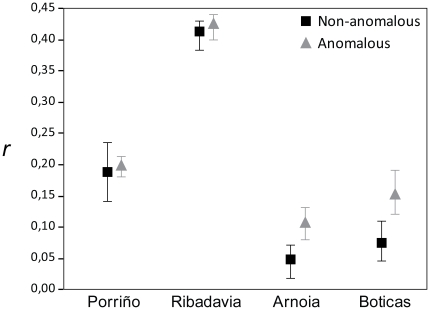
Mean relatedness for each population for anomalous and non-anomalous subsets (error bars determine the 95% confidence interval by bootstrap resampling).

We found higher relatedness values in the anomalous subset for two populations (Arnoia and Boticas) (Figure 3). Interestingly, no differences were found between subsets in populations likely affected by pollution (Porriño and Ribadavia).

None of the seven microsatellites analyzed in this study showed significant association with individual phenotypes across the seven Iberian populations (*p*>0.1). However, the chance of identifying loci associated with phenotypic traits increases with the number of unlinked markers surveyed, and our attempt for association mapping included only a few available microsatellites.

### Genetic-environmental influence on carapace anomalies

**Figure 4:**
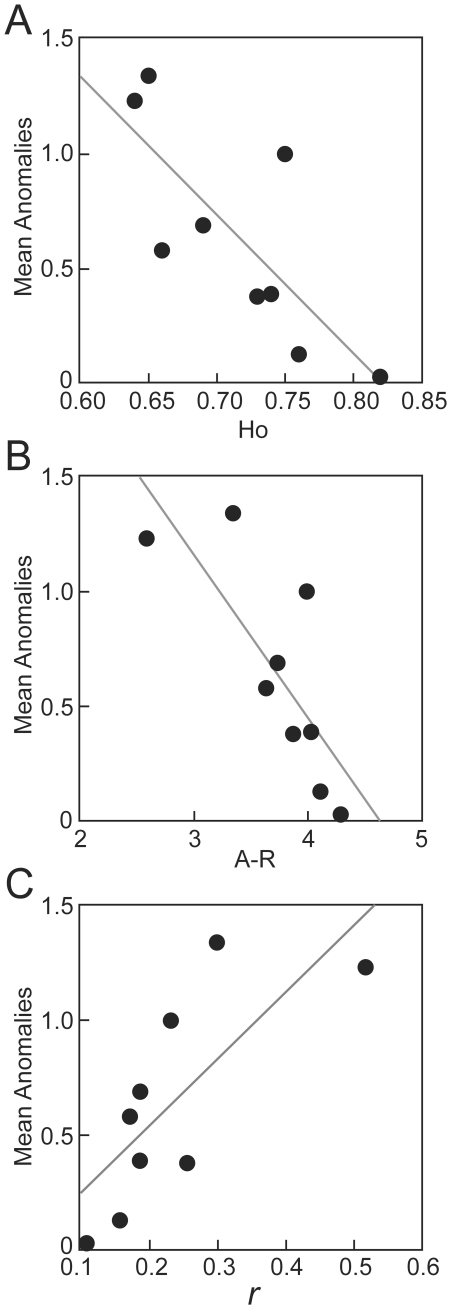
Linear regressions showing the relationship between genetic diversity indeces and mean anomalies (3A, Ho = observed heterozygosity; 3B, A-R = Allelic richness, and 3C, *r* = relatedness).

Genetic measures were strong predictors of the mean proportion of anomalies across the Iberian peninsula: observed heterozygosity, Ho (Linear Regression: F_7,1_ = 12.08, r^2^ = 0.63, p = 0.010, Fig. 4A); relatedness, *r* (F_7,1_ = 10.15, r^2^ = 0.59, p = 0.015, Fig. 4B), and allelic richness, A-R (F_7,1_ = 12.46, r^2^ = 0.64, p = 0.009, Fig. 4C). In contrast, none of the examined geographic and environmental factors were significant predictors of anomalies: latitude (Linear Regression: F_7,1_ = 3.21, r^2^ = 0.31, p = 0.153), minimum temperature (F_7,1_ = 0.94, r^2^ = 0.12, p = 0.363), precipitation (F_7,1_ = 3.996, r^2^ = 0.36, p = 0.085), and maximum temperature (F_7,1_ = 4.71, r^2^ = 0.40, p = 0.066); although the last two factors need further investigation given the low p-values. In our GLMs, each including one genetic index and the four environmental variables, genetic indices were the only significant factors. Trends found for genetic indices influencing mean anomalies hold after excluding cross correlation among environmental factors using Principal Components in GLMs [Appendix 1: Ho (*β* = −9.837, p = 0.001), A-R (*β* = −0.850, p = 0.052), *r* (*β* = 2.864, p = 0.027); environmental factors showing a non-significant effect in all models.

### Experimental incubation survey

Overall, 59.0±8.1% of hatchlings (N = 28 clutches) were symmetric under humid conditions and 65.1±6.9% under dry conditions (N = 30 clutches; Table 2). We analysed the effects of *year*, *population*, *maternal symmetry* and *treatment* on the number of symmetric hatchlings, using a GLM with binomial errors and logit link, and estimating the dispersion parameter (1.47) from the residual deviance. The model is significant (deviance ratio = 4.69, p<0.001), but the only significant factor was *population*, due to the difference between Ribadavia and Arnoia populations (t_52_ = −2.33, p = 0.024). Thus, different incubation conditions did not affect hatchling anomalies.

The duration of embryonic development, from egg-laying to hatching, ranged from 61 to 69 days. Eggs incubated in the humid treatment hatched three days earlier (range 1–4, Table 3). The duration of incubation was significantly affected by *treatment* (ANOVA, F_1,134_ = 24.94, p<0.001), *year* (F_1,134_ = 27.73, p<0.001) and *population* (F_3,134_ = 6.44, p<0.001). The average hatching success was 58.7±6.4% under humid conditions and 72.7±5.4% under dry conditions (N = 30 clutches; Table 3). We tested the effects of *year*, *population, maternal symmetry* and *treatment* on hatchling success using a GLM with binomial errors and logit link. No significant effects were found, but treatment had a marginal effect (t_53_ = −1.75, p = 0.085).

**Table 3:** Incubation period (days from egg-laying to eclosion), hatchling weight, body length (caparace length, CL), hatching success and proportion of symmetric individuals of experimental clutches, in function of *year*, *population* and *treatment* (humid/dry).

		Population			
		Arnoia	Boticas	Porriño	Ribadavia
Treatment	Year	Duration of incubation (days)			
Wet	2006	61.0±0.91 (4)	61.4±0.85 (25)	65.0±0.82 (4)	
	2007	62.3±0.67 (3)	63.4±0.64 (20)		65.4±1.34 (10)
Dry	2006	63.0±0.58 (3)	64.0±0.36 (26)	65.3±0.48 (4)	
	2007	63.8±2.56 (4)	66.5±0.61 (29)		69.3±0.93 (15)
		**Hatchling weight (g)**			
Wet	2006	3.7±0.14 (4)	4.8±0.07 (25)	5.2±0.07 (4)	
	2007	3.5±0.20 (3)	5.2±0.11 (20)		4.3±0.18 (9)
Dry	2006	3.6±0.12 (3)	5.0±0.07 (26)	5.3±0.08 (4)	
	2007	4.0±0.14 (4)	5.3±0.07 (29)		4.6±0.10 (14)
		**Hatchling size (CL, mm)**			
Wet	2006	24.0±0.46 (4)	25.9±0.21 (23)	27.3±0.33 (4)	
	2007	23.9±0.47 (3)	27.8±0.24 (18)		25.8±0.29 (8)
Dry	2006	22.8±0.41 (3)	26.4±0.22 (25)	27.4±0.41 (4)	
	2007	24.8±0.31 (29)	27.5±0.22 (29)		25.6±0.29 (11)
		**Hatching success (proportion)**			
Wet	2006	0.75±0.25 (2)	0.71±0.12 (10)	1 (1)	
	2007	0.50±0.50 (2)	0.54±0.11 (11)		0.42±0.07 (5)
Dry	2006	0.67±0.33 (2)	0.81±0.11 (10)	1 (1)	
	2007	0.60±0.40 (2)	0.70±0.07 (11)		0.64±0.09 (5)
		**Symmetric hatchlings (proportion)**			
Wet	2006	0.17±0.17 (2)	0.78±0.11 (9)	0 (1)	
	2007	0.83±0.17 (2)	0.73±0.12 (10)		0.10±0.10 (5)
Dry	2006	0.75±0.25 (2)	0.69±0.12 (10)	0 (1)	
	2007	1.00±0.00 (2)	0.75±0.10 (11)		0.32±0.18 (5)

Means are presented with standard error (SE) and sample size (number of hatchlings, except for proportions where N indicates the number of clutches). Results for Porriño and Arnoia populations should be taken with caution due to the low number of clutches studied.

Our treatment had a significant effect on hatchling weight (Table 3). Hatchlings were 4% heavier when raised under dry conditions, [4.9±0.06 g (N = 77)] than under humid conditions [4.7±0.08 g (N = 61)]. Nonetheless, no significant effect among treatments existed for carapace length [dry: 26.5±0.18 (N = 72), humid: 26.2±0.20 (N = 56)]. An ANOVA with hatchling weight as the response variable, *year* as random effect, and *population* and *treatment* as fixed factors, showed significant effects of *population* (F_3,132_ = 62.94, p<0.001) and *treatment* (F_1,132_ = 7.02, p = 0.009), but no effect of *year* (F_1,132_ = 0.00, p = 0.953) on weight. Carapace length was affected by *year* (F_1,122_ = 13.61, p<0.001) and *population* (F_3,122_ = 44.46, p<0.001) but not by *treatment* (F_1,122_ = 0.05, p = 0.821).

## Discussion

### Genetic and environmental determinants

Temperature and precipitation vary widely across Iberian populations, with drier and warmer climates in the south (Doñana and Almograve) and wetter and colder climates in the north (Porriño, Arnoia, Ribadavia and Boticas; Table 1). Our results indicate that these environmental factors do not explain the proportion of scute anomalies found in Iberian populations of *E. o. fritzjuergenobsti* (but precipitation should be analysed in future studies). In contrast, our study shows a significant relationship between population genetic diversity and scute anomalies (Figure 4). Our results corroborate previous studies in snakes that detected a negative correlation between scale anomalies and population heterozygosity [9], [10] or between fluctuating asymmetry and genetic diversity at the population level in other vertebrates [48], [49], [50], [51]. Indeed, population bottlenecks and genetic drift in small populations reduce genetic diversity and increase inbreeding. Thus, recessive alleles at loci affecting development can become fixed in populations [52], and partially explain the origin of scute anomalies in turtle populations.

Moreover, anomalous individuals from two out of the four populations studied are more related than non-anomalous individuals, suggesting heritability of this trait (Figure 3). However, we found no differences in Porriño and Ribadavia populations, but this might be explained by the potential effects of pollution increasing the proportion of anomalous individuals because these are the only studied populations surrounded by industrial areas.

### Bottleneck events in Iberian populations of E. orbicularis

Inbreeding plays an important role in the development of anomalies in wild (amphibians, [53]; lizards, [54]; and mammals, [55]) and captive populations (*Gopherus agassizii*, [56]; [57]). Consecutive bottleneck events promote inbreeding in both wild and captive populations and in some cases, severe loss of genetic diversity, which could be a main cause of anomalies [10]. However, certain species and populations might have faced bottleneck events at different times: recent populations derived from post-glacial expansions have likely experienced several bottlenecks as a result of consecutive founder events. In addition, bottlenecked populations might result from more recent fragmentation and isolation events due to environmental or anthropogenic causes. The Pleistocene refugium for *E. o. fritzjuergenobsti* was probably in northern Morocco [20], [21], suggesting a southwestern-northern colonization path on the Iberian peninsula. Hence, Iberian populations might have suffered from genetic bottlenecks twice: first, during the colonization of Iberian Peninsula from the African refugium, and second, due to more recent extreme fragmentation and isolation among the Iberian populations [58]. BOTTLENECK and *M*-ratio tests are useful for detection of recent bottlenecks but fail to detect earlier bottleneck events [33], [59]. Thus, signals of previous bottlenecks that occurred due to historical migration events giving origin to Iberian populations may not be evident in these results.

The expected loss of genetic variability and potential inbreeding in a bottlenecked population is not always uncovered by genetic surveys [60], [61]. In turtles, reduction in population size is not always reflected in the genetic structure of the species, and some species retain relatively high values of genetic diversity even when small populations persist in the face of habitat destruction and fragmentation [20] [62], [63]. An explanation for this observation was proposed by Kuo and Janzen [64], who suggested that population bottlenecks produce different genetic signatures in long-lived and short-lived species. The life history of long-lived species masks the accelerated rate of genetic drift, making it more difficult to detect recent bottlenecks by loss of genetic variation. *Emys orbicularis*, which can live up to 60 years, is an example of this long-lived species group and the lack of evidence for recent bottlenecks in the small Porriño population, even after dramatic demographic declines [65], confirms the complexity of detecting recently bottlenecked populations in this group. Conversely, the evidence of bottlenecks in Madrid, Boticas and Doñana populations could be the result of a fragmentation-isolation event which occurred only a few generations ago. Thus, these results should be interpreted with caution and we cannot discard that successive bottlenecks in our sampled populations have not contributed to increases in scute anomalies in *E. orbicularis*.

### Moisture effects during embryonic development

Our experimental incubation of clutches from four NW Iberian populations of *E. orbicularis* did not support the hypothesis that lower moisture conditions during certain stages of development result in an increase of hatchlings with scute anomalies [5], and corroborated other studies excluding moisture as a significant factor on scute anomaly formation [12], [13]. However, turtle eggshell structure varies considerably and two main functional egg types have been described: flexible-shelled eggs (lightly calcified) and hard-shelled eggs (heavily calcified) [11]. Water exchange and conductance with environment is lower in species with hard-shelled eggs, such as *Emys orbicularis*. Hence, this species might be less susceptible to environment water conditions during egg incubation, and consequently, hatchling quality little affected. The few studies on hard-shelled turtle eggs in the literature dealing with moisture effects on hatchling phenotype did not detect (*Natator depressus*, [12]) nor reported (e.g. *Chelodina expansa*, [66], [67]) significant effects of moisture on the occurrence of hatchling abnormalities, while numerous studies on flexible-shelled turtle eggs showed contrasting outcomes when suboptimal moisture levels were assessed during incubation. First, an increase of abnormality frequency on turtle carapace (*Chrysemys picta* and *Chelydra serpentina* [5]); second, an absence of moisture effects on the occurrence of anomalies (e.g. *Graptemys ouachitensis* and *G. pseudogeographica*, [13]); and third, an effect on hatchling mass/length or incubation length but no effect on hatchling abnormalities was reported (e.g. *Chelydra serpentina* [46]; *Trachemys scripta elegans*, [68], [69]; *Caretta caretta* [70]). The absence of hatchling anomalies in studies assessing the effects of moisture on hatchling phenotype during incubation, and the few studies performed in hard-shelled turtle eggs, make difficult to evaluate to what extent moisture has higher influence on hatchling abnormalities in flexible-shelled turtle eggs. However, hard-shelled turtle eggs are clearly less susceptible to environmental water deficit due to their low conduction of water and thus, drier treatments should be carried out in hard-shelled turtles in order to better evaluate the effect of water excess/deficit on hatchling phenotype during incubation. We found a higher number of anomalous hatchlings under dry treatment (65% vs. 58% under wet treatment), but only *population* explained a significant proportion of variance. Therefore, our data suggest that the effects of moisture are of minor importance in the development of these anomalies, but we cannot discount that more extreme moisture conditions may have an effect on embryos development.

In addition, the presence of populations with high proportion of anomalous individuals at higher latitudes, where temperature is lower and precipitation higher (Figure 2), suggests that moisture during incubation is likely not a constraining factor in northern populations. Soil type can also play an important role in moisture retention during egg incubation. Sandy soils are more permeable than loamy soils, and thus less water is retained to provide moisture to the eggs. Again, the suboptimal moisture hypothesis [5], [71] is not supported by soil type across Iberian populations of *E. orbicularis*, because sandy soils are characteristic of southern populations (e.g. Doñana) and loamy soils are common in the north (e.g. Porriño and Ribadavia). However, Altentejo population (SW Iberia) is the third highest anomalous population measured in this study (54% anomalous individuals) and uses sandy soils to incubate eggs, suggesting that the influence of soil type on anomaly development should be investigated further.

Both temperature and moisture may be correlated, and increased anomalies at higher temperatures have been observed experimentally in other species [72], [73]. However, Fox [4] suggested low incubation temperatures as the key factor to explain scale reductions in the Garter snake *Thamnophis elegans atratus*. We did not test temperature in our manipulative experiment because of the limited number of clutches we had to work with, although both temperature (minimum and maximum) and precipitation for each locality were considered in the bioclimatic survey.

Our experimental treatments affected the duration of incubation (three days longer on average in wet treatment) and hatchling weight (heavier in the dry treatment), but not hatchling success, indicating that incubation conditions do affect hatchling phenotype.

In summary, we do not discard the influence of moisture and temperature on embryos development, but this need to be further explored in future experiments under more extreme treatments in *Emys orbicularis*, and other chelonian taxa. Additional studies on experimental incubation of clutches from southern and eastern Iberian populations, controlling for both temperature and moisture, would be useful to disentangle the potential effects of genetics and environmental factors on scute anomalies in natural populations.

### Concluding remarks

In this study, we independently examined the potential causative factors (genetic and environmental) of scute anomalies in the turtle carapace to avoid confounding the influence of these factors. Environmental conditions may greatly affect turtle hatchling phenotypes [46], and in our experiments moisture affected hatchling weight and the duration of incubation. Nevertheless, the proportion of anomalous individuals was only related to the population of origin, suggesting intrinsic but not moisture effects on this trait.

In addition to the factors discussed above, several issues are still pending to clarify the importance of genetic and environmental factors on turtle carapace anomalies. To study the genetic implications we need to determine the heritability of scute anomalies. Although genetic-phenotype linkage could not be identified in our attempt using microsatellites, that does not imply absence of heritability, but merely lack of linkage between the relatively few microsatellites analyzed and the genomic regions responsible for this trait. In addition, hatchling anomalies were not significantly affected by maternal anomalies, but to accurately estimate heritability of this trait we also need to know the paternal phenotypes. Another important issue is to measure the influence of pollutants [6], [7] but we do not have data on levels of pollution from any of the sampled populations. However, the two highest anomalous studied populations (Porriño and Ribadavia) are potentially affected by chemicals released into the water from a neighbouring industrial area, but other highly anomalous populations are within natural areas far away from human development (river Arnoia and Boticas). Thus, pollution might be a likely contributing factor increasing the level of anomalous individuals in some studied populations, but not the main source of scute anomalies across the populations of *E. orbicularis* we sampled. Other environment stressors have also been suggested to explain fluctuating asymmetry in vertebrates (e.g. diseases, [74]; nutritional stress, [75]; or habitat disturbances, [3]; [76]) and these might also cause carapace scute anomalies in chelonians.

Our study points out to a genetic factor involved in scute anomalies rather than discarding environmental factors. Genetic factors that contribute to developmental instability can occur through a number of mechanisms: i) loss of genetic diversity, ii) degree of protein heterozygosity, iii) co-adapted gene complexes disrupted by hybridization, iv) directional selection, and v) mutant genes [77]. In this study we may exclude the effect of hybridization since the populations we studied here belong to the same *Emys obicularis* lineage that resulted from a rapid colonization of the Iberian peninsula originating in North Africa, but we do provide evidence that measurable genetic diversity is correlated with the proportion of anomalies in natural populations.

The genetic contribution to carapace anomaly formation is likely not equal across species. Comparative studies on turtle anomalies must be done on species sharing similar ecological niches and evolutionary histories to address the genetic influence among turtle species. The Iberian peninsula offers an ideal scenario, because it also harbors a second freshwater turtle, the Mediterranean pond turtle, *Mauremys leprosa*, which is sympatric with *E. orbicularis* [78]. The two species also share similar evolutionary histories with a North African glacial refugium and secondary colonization of the Iberian peninsula [79]. We found lower proportions of carapace anomalies in *Mauremys* compared to *Emys* individuals across five sympatric Iberian localities [24]; these differences may be explained, at least in part, by differences in genetic diversity, and obviously by species identity.

The challenge of identifying genes that underlie phenotypic variation in natural populations is still one of the major objectives in evolutionary biology, and very few studies have demonstrated the link between genetics and phenotypic traits [80]. We argue that genetic factors play an important role in the origin of anomalies in wild turtle populations and might serve as an indirect estimate of fitness in natural populations, but many factors clearly influence embryonic development and thus, disentangling what factors influence the occurrence of carapace scute anomalies in wild populations requires further studies using integrative approaches. Future work in *Emys orbicularis* should focus on the role of chemicals and pollution and studies of the heritability of scute anomalies to further elucidate the complexity of anomalies formation in natural populations.

## Supporting Information

Table S1 Generalized linear Models evaluating, in turn, the relative strength of each genetic index and environmental factors on mean anomalies. Here, cross-correlated environmental factors (Tmin, Tmax, Rainfall, and Latitude) were reduced into one variable using Principal Components.
(DOC)
