# Supplementary material for: Turtle Carapace Anomalies: The Roles of Genetic Diversity and Environment
Source: PLoS One. 2011 Apr 12;6(4):e18714. doi: 10.1371/journal.pone.0018714 (PMC3075271; doi:10.1371/journal.pone.0018714)
Supplement: Table S1 — Generalized linear Models evaluating, in turn, the relative strength of each genetic index and environmental factors on mean anomalies. Here, cross-correlated environmental factors (Tmin, Tmax, Rainfall, and Latitude) were reduced into one variable using Principal Components. (DOC) [file pone.0018714.s001.doc]

**Table S1.** Generalized linear Models evaluating, in turn, the relative strength of each genetic index and environmental factors on mean anomalies. Here, cross-correlated environmental factors (Tmin, Tmax, Rainfall, and Latitude) were reduced into one variable using Principal Components.

| **Term** | ***Estimate*** | ***SE*** | ***L-R χ2*** | ***P*** |
| --- | --- | --- | --- | --- |
| Intercept | 7,642 | 2,236 | 7,484 | 0,006 |
| HO | -9,837 | 3,119 | 6,698 | 0,009 |
| Environment PC1 | -0,141 | 0,110 | 1,492 | 0,222 |
| HO*Environment PC1 | -0,486 | 1,038 | 0,216 | 0,641 |
|  |  |  |  |  |
| Intercept | 3,889 | 1,585 | 4,609 | 0,032 |
| AR | -0,849 | 0,393 | 3,761 | 0,052 |
| Environment PC1 | -0,033 | 0,114 | 0,084 | 0,771 |
| AR*Environment PC1 | 0,151 | 0,237 | 0,392 | 0,531 |
|  |  |  |  |  |
| Intercept | -2,38E-05 | 0,239 | 9,87E-09 | 0,999 |
| R | 2,864 | 1,128 | 4,861 | 0,027 |
| Environment PC1 | 0,054 | 0,072 | 0,556 | 0,455 |
| R*Environment PC1 | -0,459 | 0,696 | 0,426 | 0,514 |
